# Relationships in oocyte recipient couples – a Swedish national prospective follow-up study

**DOI:** 10.1186/1742-4755-11-38

**Published:** 2014-05-26

**Authors:** Gunilla Sydsjö, Claudia Lampic, Marie Bladh, Agneta Skoog Svanberg

**Affiliations:** 1 Obstetrics and Gynecology, Department of Clinical and Experimental Medicine, Faculty of Health Sciences, Linköping University, Linköping, Sweden; 2 Department of Neurobiology, Care sciences and Society, Karolinska Institutet, Stockholm, Sweden; 3 Department of Women’s and Children’s Health, Uppsala University, Uppsala, Sweden

**Keywords:** Couples relationship, Oocyte, Donation, Infertility

## Abstract

**Background:**

The long-term effect of treatment with donated oocytes on women’s and men’s perception of their relationship has been little studied. Thus the aim of this study was to analyse satisfaction with relationships in couples at the time of acceptance for treatment and 2–5 years after treatment with donated gametes and to compare them with IVF couples treated with their own gametes.

**Method:**

A prospective follow-up study in which data were collected twice on two groups; cohabitating couples receiving oocyte donation and cohabitating IVF couples using their own gametes. A standardised instrument, the ENRICH inventory, was used to gain information about the couples’ subjective experience of their relationships.

**Results:**

At acceptance for treatment the couples in the two groups assessed their relationships as being very solid on all dimensions and that the women receiving treatment with oocyte showed a higher satisfaction compared to women treated with own gametes. For couples that did have a child, the group of women who had been through the oocyte donating program reported a better quality of their relationship than women in the control group. There were no significant differences in perceived relationship quality between men in the different groups, whether they had a child or not.

**Conclusions:**

From a long-term perspective couples using oocyte donation treatment have a balanced and solid view of their relationship and treatment, having children or not after treatment did not affect the nature of the relationships.

## Background

Studies on how couples who are treated with IVF adjust and perceive their relationship are in agreement that the couples appear for the most part to handle their relationship and adjustment before and after treatments well [1-3].

Couples in which the woman has not been able to get pregnant and have a child after IVF treatment also seem to handle their relationship in a balanced way [2]. In a long term follow up of 788 individuals, we found that men and women who were living together 20 years after IVF treatment had been terminated assessed their relationship as good, whether the couples had become parents or not [4]; most of the couples, i.e., 90%, were living or had been living with children in their family. The effect on marriages and the opinion on the relationship are of clinical interest since many couples are worried about the effects of infertility on sexuality, closeness, communication and future plans.

To be diagnosed with ovarian insufficiency or having been treated for cancer with chemotherapy and therefore being unable to have children with one’s own oocytes will put most women under considerable stress and force them to start a decision process on how to have a family. Adoption, oocyte donation or embryo donation or surrogacy might be the choices these women and their partner have to face. For some women, awareness of the inability to conceive might have been lifelong, which is the case for a women diagnosed with Turner syndrome, but for other women ovarian insufficiency is detected only during an infertility investigation and is therefore shocking and represents threatening information affecting the individual’s self-esteem and psychological health.

For some women, biological age is an important factor when they are trying to have children and for them oocyte or embryo donation is a solution. A woman age 40 or older faces other problems that might make her more aware of the couple’s situation and the stability in their relationship, problems such as age, going through menopause at the time of becoming pregnant, and having a child. The psychological burden of infertility varies and different studies of mental health and wellbeing in general and, for example, of assessment of quality of life show different results [5-8].

In 2003 when egg donation with identifiable donors was first allowed in Sweden we started a national study including all centres that were allowed to perform IVF treatment with donated gametes i.e., the seven university hospitals in Sweden. In Sweden the indication for treatment with donated oocytes must be medical and the receiving women are supposed to be of fertile age so most clinics do not treat women over 40 years of age.

The long term effect of treatment with donated oocytes on women’s and men’s perception of their relationship in a national sample as well as analysis of the subjective bonding/affection effect of having a child after oocyte donation have not been previously studied to the best of our knowledge. The aim of the present study was thus to study relationships quality in couples who were scheduled for IVF treatment with donated oocytes at the start of and 2–5 years after treatment compared to couples undergoing IVF with their own gametes.

## Materials and methods

### Procedures

*The Swedish study on gamete donation* is a prospective longitudinal study of all donors and recipients of donated gametes. The multicentre study includes subjects from all infertility clinics performing gamete donation in Sweden, clinics located at the University hospitals in Stockholm, Gothenburg, Uppsala, Umeå, Linköping, Örebro, and Malmö. During the period 2005 to 2008 consecutive samples of couples starting oocyte donation treatment were approached regarding participation and asked to complete the ENRICH (Evaluating & Nurturing Relationship. Issues, Communication & Happiness) inventory. Exclusion criteria were inability to read and write the Swedish language.

#### Oocyte recipient couples

For oocyte receiving couples a total of 477 individuals were invited to participate and of these 106 declined participation, 45 were excluded and 24 did not respond leaving 302 individuals in the study. However, for the purpose of this study only participants where both partners in the couple had responded completely to the ENRICH inventory at first assessment and who still lived with the same partner as when starting treatment were included leaving 240 individuals (120 couples) for analysis. A total of 104 individuals (52 couples) have given complete answers to the ENRICH inventory at both assessments.

#### Comparison group

For controls we used IVF couples using their own gametes and treated during the same time period (2005–2008) at the clinics in Linköping, Örebro, Uppsala, Gothenburg and Umeå. This comparison group was created to be used in different reports and comparisons in this national long term follow up study. A consecutive sample of 212 eligible heterosexual couples (424 individuals) starting assisted reproduction who all could read and write Swedish were approached for study participation. Of these 151 couples accepted participation and individually completed the ENRICH inventory at the start of treatment.

A total of 238 individuals (119 couples) have answered the ENRICH inventory at first assessment. However, for the purpose of this study only participants where both partners in the couple had responded completely to the ENRICH inventory at first assessment and who still lived with the same partner as when starting treatment were included, in total 238 individuals (119 couples). A total of 122 individuals (61 couples) have given complete answers to both assessments of the ENRICH inventory.

#### Clinical practice

Couples requesting IVF treatment with own or donated gametes have to have a stable relationship defined as having lived together for the two years before the start of treatment. This recommendation is practise at all the participating clinics. Furthermore, the indications for treatment with oocytes at the clinics are: Turners syndrome, ovarian insufficiency, or earlier chemotherapy treatment. A psychological and medical interview is performed with both the man and the woman. This is done by a psychologist and the responsible IVF doctor. They have to meet the criteria of being healthy both somatically and mentally i.e., no severe illness. The couple must also understand and agree to the law that states that the child resulting from oocyte donation has the right to get identifying information about his or her donor when reaching mature age, i.e. around 18 years. Most clinics offer two to three treatment cycles, financially covered by the county where the couple lives. There were different waiting times at different centres because of a lack of oocyte donors. The waiting time from acceptance to treatment could vary between 6 months up to two years.

#### First assessment (T1)

In general, the couples received oral information about the purpose of the study from their doctor at their own University clinic. They were also given written information. The couples were asked to answerer the ENRICH inventory separately at the clinic or at home, if they preferred.

#### Second assessment (T2)

The second assessment with the ENRICH inventory was due around 2–5 years after the first assessment. In this part of the study the couples all received an information letter and the ENRICH inventory was sent by post to the men and women separately.

The study was approved by the Regional Ethical Review Board in Linköping.

#### Measures

##### The ENRICH inventory

We used the Swedish version of the ENRICH marital inventory, originally created by Olson and co-workers [9], to study marital/partner relationship functions and dynamics. The instrument provides scores of each partner’s evaluation of their relationship as they assessed their present relationship in 10 categories comprising 10 items each. The ENRICH different categories can be described as follows:

Personality Issues: Examines an individual’s satisfaction with his or her partner’s behaviours.

Communication: Is concerned with an individual’s feelings and attitudes toward communication in the marriage. Items focus on the level of comfort felt by the respondent sharing and receiving emotional and cognitive information from the partner.

Conflict Resolution: Assesses the partner’s perception of the existence and resolution of conflict in the relationship. Items focus on how openly issues are recognized and resolved, as well as the strategies used to end arguments.

Financial Management: Focuses on attitudes and concerns about the way economic issues are managed within the marriage. Items assess spending patterns and the manner in which financial decisions are made.

Leisure activities: Assesses preferences for spending free time and leisure time. Items reflect social vs. personal activities, shared vs. individual preferences, and expectations about spending leisure time as a couple.

Sexual Relationship: Examines the partner’s feelings about the affectionate and sexual relationship. Items reflect attitudes about sexual issues, sexual behaviour, and sexual fidelity.

Children and parenting: Assesses attitudes and feelings about having and raising children. Items focus on decisions regarding discipline, goals for the children, and the supposed impact of children on the couple’s relationship.

Family and Friends: Assesses feelings and concerns about relationships with relatives, in-laws, and friends. Items reflect expectations for and comfort with spending time with family and friends.

Egalitarian Roles: Focuses on an individual’s feelings and attitudes about various marital and family roles. Items reflect occupational, household, sex, and parental roles. High scores indicate a preference for more egalitarian roles.

Conception of Life: Examines the meaning of values, religious beliefs and practice, and conception of life within the marriage.

A score of 50 is the most positive outcome and each category scale can vary between 10 and 50 points. There are six options for each item ranging from “in total agreement” to “do not agree at all”. The summed category scale scores provide a global assessment of marital satisfaction varying between 100 and 500 points.

We have also used the Positive Couple Agreement (PCA) scale, which was derived from the ENRICH subscales. This PCA scale score is acquired through the couples’ agreement in describing their relationship in positive terms on each question for each scale. This results in a measurement ranging from 0% to 100% agreement, depending on the number of agreements and the total number of questions in each subscale.

The ENRICH subscales have shown good internal consistency (alpha, range = .69-97) and test-retest reliability (r_tt_, range = .65-.94) as well as content and construct validity [9]. The discriminate and concurrent validities of these scales have been proven [10]. Wadsby [11] have evaluated the Swedish version of the inventory and found the reliability and the validity of the instrument to be acceptable [11]. As a check on the quality of the ENRICH inventory for this study Cronbach’s alpha was evaluated for the total scores and was found to be 0.900 for oocyte recipients and 0.879 for couples treated with traditional IVF.

#### Statistics

Demographic differences between the two groups were evaluated using Pearson’s Chi-square test or Fisher’s Exact Test if cell count was below 5. The ENRICH scores (i.e., the ten factors as well as the total scores on both occasions) for the study group were tested for normality by use of the Kolmogorov-Smirnov test. The data were also examined visually by scatter-plots to identify possible extreme values. As the assumption of normality could not be met for all of the variables studied, we chose to use a non-parametric approach when analysing the data for each time-point. The Mann–Whitney *U* test was used to examine differences between the groups, “oocyte recipients” and “traditional IVF” as well as “no children through treatment” and “children through treatment. For the study of changes in scores over time Wilcoxon Paired Signed Rank Test was used. All statistical analyses was performed using IBM SPSS version 20.0 (Armonk, NY, USA). A p-value < 0.05 was considered statistically significant.

## Results

Demographic factors such as age, level of education and number of previous children as well as children after treatment are displayed in Table 1. No differences were found between the groups. During the study period 50 (61%) couples among the oocyte recipients and 41 (46%) of the couples among the IVF treated couples had become parents (p = 0.051).

**Table 1:** Demographic data for women and men participating in the study

		**Oocyte recipients**		**Traditional IVF**		
		**n**	**%**	**n**	**%**	p-value*
**Women**						
Age	≤ 30	29	24..2	41	34.5	0.081
	> 30	91	75.8	78	65.5	
Education	Elementary	11	9.2	4	3.4	0.174
	High school	44	37.0	46	38.7	
	University	64	53.8	69	58.0	
Biological children	No	113	94.2	107	89.2	0.161
	Yes	7	5.8	13	10.8	
Adoptive children	No	118	98.3	120	100.0	0.498
	Yes	2	1.7	0	0.0	
Step children	No	110	91.7	117	97.5	0.046
	Yes	10	8.3	3	2.5	
Child after treatment	No	32	39.0	48	53.9	0.051
	Yes	50	61.0	41	46.1	
**Men**						
Age	≤ 30	18	15.0	29	24.4	0.068
	>30	102	85.0	90	75.6	
Education	Elementary	9	7.6	10	8.5	0.840
	High school	62	52.1	57	48.3	
	University	48	40.3	51	43.2	
Biological children	No	100	83.3	109	90.8	0.083
	Yes	20	16.7	11	9.2	
Adoptive children	No	118	98.3	120	100.0	0.498
	Yes	2	1.7	0	0.0	
Step children	No	119	99.2	113	94.2	0.031
	Yes	1	0.8	7	5.8	

*Pearson’s Chi-square test. If cell count is below 5 Fisher’s exact test is used.

At acceptance for treatment the women in the oocyte receiving couples assessed their relationship as better compared to the IVF women in the dimensions “Children”, “Communication” and “Leisure”. In the “Total Score” no differences between groups were found (Table 2). At the assessment 2–5 years after treatment the women in the control group, i.e., the women treated with IVF with their own gametes, had a lower total score as well as lower scores on the dimensions “Children”, “Conflict”, “Financial” and “Leisure” compared to women receiving donated oocytes (Table 3). Moreover, oocyte receiving men scored higher on the dimension “Communication” compared to men treated with IVF.

**Table 2:** The couples’ assessment of their relationship at acceptance for infertility treatment

	**Oocyte recipients**		**Traditional IVF**		Oocyte vs. IVF*	
	**Woman**	**Man**	**Woman**	**Man**	**Woman**	**Man**
	**Mean/SD**	**Mean/SD**	**Mean/SD**	**Mean/SD**	**p-value**	**p-value**
Personality	44.1/4.9	42.4/4.5	43.4/4.6	41.7/5.1	0.112	0.379
Sexual	43.1/3.5	42.9/3.3	43.2/3.3	43.0/3.6	0.325	0.145
Children	45.1/3.1	44.4/3.3	43.8/3.9	43.7/3.4	0.038	0.095
Family	44.4/4.3	43.6/4.4	44.5/3.6	43.2/5.1	0.287	0.952
Egalitarian	40.8/3.8	41.2/3.6	40.5/3.6	41.1/3.6	0.538	0.189
Conception	40.6/3.3	39.4/3.3	39.9/3.5	39.1/4.0	0.932	0.575
Communication	44.0/4.6	43.6/4.5	43.4/4.8	42.5/5.1	0.005	0.090
Conflict	41.9/4.9	40.7/4.5	40.4/5.4	39.3/5.9	0.892	0.708
Financial	43.2/4.3	42.4/4.1	42.9/4.0	42.2/4.6	0.373	0.841
Leisure	40.8/5.4	39.0/5.3	40.6/4.5	38.1/5.7	0.029	0.450
Total	428.1/31.2	419.5/29.8	422.4/28.0	413.9/33.4	0.059	0.199

*Mann–Whitney *U*-test.

**Table 3:** The couples’ assessment of their relationship two to five years after treatment

	**Oocyte recipients**		**Traditional IVF**		Oocyte vs. IVF*	
	**Woman**	**Man**	**Woman**	**Man**	**Woman**	**Man**
	**Mean/SD**	**Mean/SD**	**Mean/SD**	**Mean/SD**		
Personality	42.7/5.5	41.7/5.6	41.9/5.8	42.2/8.5	0.479	0.554
Sexual	42.0/5.8	418/5.4	41.5/9.2	41.8/10.2	0.272	0.511
Children	41.2/7.6	38.6/6.2	38.4/6.1	37.3/6.6	0.042	0.196
Family	42.8/4.9	41.5/4.1	42.2/5.3	42.6/6.7	0.658	0.181
Egalitarian	39.3/5.2	38.4/6.3	38.4/5.8	36.8/6.7	0.327	0.220
Conception	44.4/9.4	42.5/6.0	42.2/6.0	41.4/6.1	0.256	0.282
Communication	42.0/5.3	42.0/5.1	40.6/5.1	40.2/5.5	0.122	0.049
Conflict	44.2/4.6	42.2/5.5	42.2/4.5	42.2/6.5	0.019	0.616
Financial	40.1/3.8	39.8/3.8	38.5/4.4	40.0/4.2	0.033	0.510
Leisure	39.6/4.7	38.6/4.8	37.8/4.7	37.7/4.2	0.017	0.170
Total	418.3/37.0	407.1/41.4	403.5/41.9	402.1/46.3	0.050	0.468

*Mann–Whitney *U*-test.

Over time, a decline of scores in almost all of the subscales was seen in both groups. However, increases were found on the dimensions “Conception” and “Conflict” among men and women in both groups (though not achieving statistical significance for oocyte receiving men) (Table 4).

**Table 4:** Test for difference on the ENRICH scores for each subscale comparing measurements before treatment and two to five year after treatment/childbirth within the groups

	**Oocyte recipients**		Traditional IVF*	
	**Woman**	**Partner**	**Woman**	**Partner**
Personality	0.003	0.289	0.001	0.560
Sexual	0.440	0.268	0.619	0.114
Children	< 0.001	< 0.001	< 0.001	< 0.001
Family	0.007	0.027	0.006	0.073
Egalitarian	0.016	0.002	< 0.001	0.001
Conception	0.014	0.090	0.006	0.004
Communication	< 0.001	0.007	0.003	0.005
Conflict	0.001	0.009	0.001	0.001
Financial	0.124	0.012	0.429	0.553
Leisure	0.001	0.102	< 0.001	0.027
Total	< 0.001	0.003	< 0.001	0.002

*Wilcoxon Paired Signed Rank Test.

The agreement between the men and women in the pairs was good at the first assessment but at the second assessment we found a decline in this measure in both groups (but the decline appears to be somewhat more prominent in the group of men and women being treated with their own gametes), mean decline over all scales was 6.99 for oocyte recipients and 9.55 for couples treated with their own gametes, p = 0.772 (Table 5).

**Table 5:** Test for difference on PCA-scores between the two groups and the measurements before treatment and 2–5 year after treatment/childbirth

	**Oocyte recipients**		**Traditional IVF**		**Oocyte recipients**	**Traditional IVF**
	**1st measurement**	**2nd measurement**	**1st measurement**	**2nd measurement**	p-value*	p-value*
Personality	70.6/21.2	66.2/23.7	65.4/19.9	60.8/23.8	<0.001	0.001
Sexual	85.6/17.0	72.3/25.7	84.5/17.3	65.2/27.4	<0.001	<0.001
Children	79.9/14.1	68.5/22.3	75.2/17.1	61.0/23.4	<0.001	<0.001
Family	76.4/18.2	70.2/19.6	75.5/17.9	69.3/19.6	<0.001	0.002
Egalitarian	68.8/13.3	61.9/17.3	61.5/15.7	57.2/20.4	<0.001	<0.001
Conception	67.4/11.6	63.1/17.9	65.2/15.2	57.9/17.7	0.001	0.001
Communication	76.9/19.0	69.6/23.4	72.7/21.1	60.3/27.6	<0.001	<0.001
Conflict	63.2/18.7	56.0/23.4	57.6/21.9	44.9/27.2	<0.001	0.001
Financial	73.2/16.8	69.0/17.3	69.3/18.5	67.2/24.2	<0.001	0.152
Leisure	57.9/22.7	53.3/24.3	57.6/21.7	45.2/26.1	0.001	<0.001

*Wilcoxon Paired Signed Rank Test.

Oocyte receiving women who had been successful in getting pregnant and had had a child rated their relationship quality as better than did comparison women who became pregnant and had a child with their own gametes (Table 6). For couples that did have a child, the group of women who had been through the oocyte donating program were assessed as having a better quality of their relationship than women in the control group. There were no significant differences in perceived relationship quality between men in the different groups, whether they had a child or not.

**Table 6:** Test for difference on the ENRICH scores for each subscale comparing measurements before treatment and two to five years after treatment/childbirth reported by type of treatment and child/no child after treatment*

		**Oocyte recipients**		**Traditional IVF**					
		**No child after treatment**	**Child after treatment**	**No child after treatment**	**Child after treatment**	**p-value**^**a**^	**p-value**^**b**^	**p-value**^**c**^	**p-value**^**d**^
Woman	Total 1st measurement	425.2/24.0	433.4/29.2	421.4/30.7	422.0/28.4	0.039	0.856	0.992	0.025
	Total 2nd measurement	410.3/43.9	414.0/41.5	418.1/36.9	393.3/40.6	0.710	0.013	0.472	0.012
Man	Total 1st measurement	417.6/23.6	425.8/27.7	413.4/35.1	416.6/32.6	0.099	0.717	0.873	0.121
	Total 2nd measurement	400.9/41.8	402.3/49.8	410.3/49.7	395.2/44.2	0.552	0.301	0.530	0.324

*Mann–Whitney *U*-test.

^a^Comparison of no child after treatment vs child after treatment among oocyte recipients.

^b^Comparison of no child after treatment vs child after among traditional IVF.

^c^Comparison of oocyte vs traditional IVF among those with no child after treatment.

^d^Comparison of oocyte vs traditional IVF among those with child after treatment.

## Discussion

Couples who have been treated with donated oocytes and were still cohabiting some years after treatment showed a stable and balanced relationship over time. Being childless after treatment or having a child after treatment did not have a negative effect on the couple’s assessment of the relationship. A decline in ENRICH scores over time could be seen in the oocyte receiving group as well as in the comparison group of women and men using standard IVF-treatment.

This is accordance with our earlier findings on IVF couples relationship after both successful and an unsuccessful treatment [2-4]. One explanation could be that the couples should have had a stable relationship before treatment. For the couples with an unsuccessful treatment there might be hope for future successful treatments or adoption of a child and that could have a positive effect on their relationship at this point [2].

One limitation of the present study is that it is restricted to investigation of the development of relationships among couples that enrolled and remained in the study and had the same partner at follow-up. Unfortunately, we have no information about the reasons for separation among those couples not longer living together. Future follow-ups will perhaps give us information about this group. However, the selection bias could be seen as equivalent in both groups and should have no impact on group comparisons. One aspect that may have had an impact on the present results is the fact that study participants were recruited shortly after oocyte donation treatment was allowed in Sweden. Another limitation affecting the results might also be that we have only used the ENRICH to assess the quality of the relationship–it would have been a strength to also interview the men and women in order to gain additional understanding of the individuals’ opinion on the relationship–even though the use of such methods is also hampered by social desirability effects. A further limitation is that we have no information about the length of the participating couple’s relationship before treatment or the length of the relationship for couples that have decline participation. We are also unable to evaluate whether there are differences between men and women and their perceived relationship quality based on whether the infertility is male factor, female factor, or combined.

The main strength of this study is that it is a national study cohort and that we have been able to follow the groups over a long period of time. All couples who have been treated have been supported by the national health insurance program, so selection bias related to financial ability to pay for treatment is unlikely.

We have not measured “quality of life” and the effects of the inability to get pregnant nor have we studied how having a child may have affected the couple’s quality of life and mental health in relation to their opinion on their relationship, effects that might influence the quality of the relationship. In the review by Chachamovich and colleagues [12] the results from the 14 studies included showed that men who had a poor marital relationship displayed lower mental health scores [12]. Women on the other hand that showed lower scores on mental health, social functioning and emotional behavior and had significantly lower scores in several domains compared to the men indicating that infertility has more negative effect on women at least when mental health and social functioning are assessed.

We were not able to see any negative effects on the opinion on sexuality in our study, neither the men nor the women expressed problems in this area. For women in the present study climacteric problems might be of concern as found in the Carter study but we have not studied this specifically but we were not able to detect such an effect on, for instance, sexuality [7]. The couples also rated their communication as good even though we found a decline in the follow up. Schmidt and co-worker’s found that both men and women who reported communication problems at the beginning of infertility treatment showed more fertility stress at the follow up 12 months post treatment [13]. Coping strategies such as “Active-avoidance coping” (e.g., avoiding being confronted with pregnant women or children and using work an avoiding action) was a significant predictor of high fertility problem stress [13,14]. The same research group reported that among men and women not becoming pregnant the 25.9% and 21.1% reported high martial benefit of the infertility experiences [15] The study by Carter et al. [7] with the aim of examining the emotional, sexual, physical, and quality-of-life (QOL) impact on infertile women on the women awaiting treatment with donated oocytes showed that depression was frequent and that 59% of the women had high levels of distress and that the women were affected with sexual dysfunction [7]. Relationship satisfaction measured by the Abbreviated Dyadic Adjustment Scale the scores were comparable to the population norm. The women showed good physical QOL but below average on the mental part of the QOL (Medical Outcomes SF-12 Health Survey). The results also revealed that the recipients had concerns about the long-term effects of the treatment. Sexual problems might have a negative effect on the couple’s relationship in the long term and there are several studies that have reported on sexual dysfunction being more prevalent in female partners of infertile couples. It has also been reported that female sexual function is positively correlated with male partner sexual function in this population [16].

The birth of a child has an effect on most couple’s satisfaction with their relationship and mostly there is an overall decline [17]. In this study the effect of having a child as expressed couples’ opinions of the quality of their relationships was most positive for the couples in which the woman became pregnant with donated gametes. This positive result indicated that at least the couples who participated in this study were not negatively affected by the birth of a child not genetically connected with the woman given birth. The length of waiting time and preparation before treatment might have influenced this outcome.

Future research is needed on the couples that have separated and the dropouts after acceptance for treatment in order to further understand the needs these couples might have for help from the professions when starting treatment and also to gain more knowledge on the different kinds of family constructions and the effect of this on the quality and stability on the marital relationship.

## Competing interest

The author declared that they have no competing interest.

## Authors’ contribution

GS, CL, ASS, planned and designed the study. GS, ASS, MB, and CL, contributed to the acquisition of data. All authors have been responsible analysing the data and for writing the paper. All authors were involved in drafting/revising of the paper and approved the final version of the manuscript for submission.
